# Supplementary material for: STRAIGHTMORPH: A Voice Morphing Tool for Research in Voice Communication Sciences
Source: Open Res Eur. 2025 Jan 10;4:154. Originally published 2024 Jul 24. [Version 2] doi: 10.12688/openreseurope.18055.2 (PMC11736103; doi:10.12688/openreseurope.18055.2)
Supplement: Peer review history

# Reviewer report: Reviewer response for version 2

**DOI:** 10.21956/openreseurope.20747.r49993

**Published:** 2025-03-03

**Authors:** Lee M. Miller

**Affiliations:** 1 University of California, Davis, USA

This report introduces a software tool for parametric acoustical morphing among voices with different identity or expression: STRAIGHTMORPH. It is built on Legacy-STRAIGHT, a well established and highly regarded speech analysis-synthesis (vocoder) package developed by the second author. The morphing capability of Legacy-STRAIGHT has existed for many years, but there lacked a user friendly interface to take full advantage of it. STRAIGHTMORPH supports parametric morphing along five basic acoustic dimensions (F0, spectral amplitude, aperiodicity, time, and frequency) which capture many of the attributes that convey perceptual distinctions among voices (or other complex natural sounds with pitch such as nonhuman primate vocalizations or music). Morphing along one or more of these dimensions allows the user to produce high quality voice continua, averages, and caricatures. Importantly, this allows one to dissociate perceptual changes from well-controlled acoustical changes; consequently, a primary application area is neurobehavioral studies of voice perception. This report gives step-by-step instructions on how to use the helpful GUI in a variety of use cases. It does not - and need not -  unpack the technical foundations of the analysis-synthesis in Tandem-STRAIGHT, as this is covered in numerous prior publications. All code including a tutorial function are freely available on github. STRAIGHTMORPH offers a tremendously valuable, powerful, and easy-to-use tool for a wide variety of voice-acoustic researchers.

**SPECIFIC COMMENTS **(page numbers refer to the pdf)

**MAJOR:**

- Not everyone is adept at reading spectrograms. So that readers can appreciate what the morphing is actually doing, please embed the audio for the examples shown. This would increase the clarity and impact of the report.
- A brief explanation and justification of using Legacy-STRAIGHT, as opposed to TANDEM-STRAIGHT (or WORLD, for that matter), would be welcome. Why not use TANDEM-STRAIGHT, which was developed as an improved successor to Legacy-STRAIGHT?
- p5. I find the term “morphing rate” to be very confusing, because it is in no straightforward way a “rate”. Since the values indicate the weighting of each mObject in a morph for a given parameter (F0, spectralamplitude, etc.), a more accurate term would be “morphing weight”.
- A brief passage on practical limitations would be helpful.  How different can utterances be before the morphing typically fails? How much correspondence must there be between the time and frequency anchors? What vocal attributes are NOT captured well or readily parameterizable in an mObject?

**MINOR:**

- p3. “a similar implement” –> “a similar implementation”
- p4. “Object.aperiodicityIndex containing a matrix of similar size as the spectrogram” Shouldn’t “similar” instead be “identical”?
- p3-4. Please define the terms “mObject” and “anchor” briefly upon first use in main text, rather than waiting for the full explication later.
- p4: “spoken a female” -> “spoken by a female”
- “it can be first be” -> “it can first be”
- Figure 1 legend. C: Please define blue and green lines (presumably the manual f0 range restriction).
- p5 “cell array of structure” -> cell array of structures”
- Figure 2 legend. It would help to explain briefly why the representations differ between A-C and D-E.  For instance “A-C. Smoothed spectrograms highlight coarse features such as formants.” “D-E. Unsmoothed spectrograms highlight fine structure such as harmonics and their spacing (F0)”.
- p7. “unnatural sounding stimuli...” replace ellipsis with period.
- p7. define “TVAs”.  Alternately, leave this entire sentence out, as the deep dive seems more fitting for a Discussion than a technical explication.
- p7. The term “anti-voices” makes sense, but it’s not clear why a morph between the N-average and a given mObject is an  “anti-caricature”.
- p7. “While all points of that space likely do not correspond” should read “While not all points of that space likely correspond”, or perhaps clearer, “While it is unlikely that all points of that space correspond”.
- p7 Parameter-specific morphing. You say “mRates.duration”, but on p5 the only 5 fields of mRates are listed as: F0, spectralamplitude, aperiodicity, time, and frequency. Same for “frequency” vs “anchorFrequency”. Please use the same field/parameter names throughout.

Is the rationale for developing the new software tool clearly explained?

Yes

Is the description of the software tool technically sound?

Yes

Are sufficient details of the code, methods and analysis (if applicable) provided to allow replication of the software development and its use by others?

Yes

Is sufficient information provided to allow interpretation of the expected output datasets and any results generated using the tool?

Yes

Reviewer Expertise:

Neurobiology of speech perception

I confirm that I have read this submission and believe that I have an appropriate level of expertise to confirm that it is of an acceptable scientific standard, however I have significant reservations, as outlined above.

# Reviewer report: Reviewer response for version 2

**DOI:** 10.21956/openreseurope.20747.r49705

**Published:** 2025-01-27

**Authors:** Stefan Schweinberger

**Affiliations:** 1 University of Jena, Jena, Germany

This is a crisp and clearly written paper which describes the voice morphing tool STRAIGHTMORPH – which is based on the long-standing work of the second author of the paper, and which serves the community of voice communication scientist by making high-quality software more accessible.

 This is an excellent initiative, and with its high accessibility and fine documentation, there can be no doubt that STRAIGHTMORPH will be a useful resource for the growing community of voice researchers.

 I only have a few minor points regarding this already revised version:

  

- In the first paragraph, the authors emphasize how it is important to parametrically manipulate the physical characteristics of complex naturalistic stimuli. In this context, while I believe it is adequate if stimulus examples for the software package are kept relatively simple (e.g., vowels, single words, or primate vocalizations, a few comments on how STRAIGHTMORPH can be used to morph complex naturalistic vocalisations (e.g., sentences) would be welcome, and these might enhance the impact of the software tool.
- The Section Legacy STRAIGHT is highly welcome. Two relevant references might be worth adding here: Specifically, I´m referring to the Methods Chapter “Voice Morphing” by Skuk and Kawahara (in S. Frühholz and P. Belin (Eds.): *The Oxford Handbook of Voice Perception Chapter 31, pp. 685-706*), which provides a relatively extensive discussion of voice morphing technology, and also to an early empirical paper from 2008 ( https://doi.org/10.1016/j.cub.2008.04.015 ) which could be added to the examples for how the method generated exciting results. [Schweinberger SR.et.al.2008 - Ref 1]
- At this point on p.3, please also change “an growing number of researchers” to “a growing number of researchers”
- Similarly on p4, “an time-frequency map” should read “a time-frequency map”.
- On P.7, 2 ^nd^ paragraph “Note that using mRates values too negative or too much above 1 will likely result in unnatural sounding stimuli...”: My recommendation is to omit the multiple dots at the end. If possible, be more precise as to the ways in which this may result to unnatural sounding stimuli.

P.7: a blank seems needed after the closed parenthesis in “…Latinus et al., 2013)– potentially”.

Is the rationale for developing the new software tool clearly explained?

Yes

Is the description of the software tool technically sound?

Yes

Are sufficient details of the code, methods and analysis (if applicable) provided to allow replication of the software development and its use by others?

Yes

Is sufficient information provided to allow interpretation of the expected output datasets and any results generated using the tool?

Yes

Reviewer Expertise:

Voice Perception, Person Perception, Social Interaction, Social Neuroscience

I confirm that I have read this submission and believe that I have an appropriate level of expertise to confirm that it is of an acceptable scientific standard.

## References

[1] : Auditory adaptation in voice perception. *Curr Biol* .2008;18(9) : 10.1016/j.cub.2008.04.015 684-8 10.1016/j.cub.2008.04.015 18450448

# Reviewer report: Reviewer response for version 2

**DOI:** 10.21956/openreseurope.20747.r49651

**Published:** 2025-01-22

**Authors:** Nadine Lavan

**Affiliations:** 1 Queen Mary University of London, London, England, UK

The authors have addressed my initial concern in full. I am thus happy to recommend for this manuscript to be approved.

Is the rationale for developing the new software tool clearly explained?

Yes

Is the description of the software tool technically sound?

Yes

Are sufficient details of the code, methods and analysis (if applicable) provided to allow replication of the software development and its use by others?

Yes

Is sufficient information provided to allow interpretation of the expected output datasets and any results generated using the tool?

Yes

Reviewer Expertise:

Voice perception

I confirm that I have read this submission and believe that I have an appropriate level of expertise to confirm that it is of an acceptable scientific standard.

# Reviewer report: Reviewer response for version 2

**DOI:** 10.21956/openreseurope.20747.r49723

**Published:** 2025-01-15

**Authors:** Andrey Anikin

**Affiliations:** 1 Univ. of Lund, Lund,, Sweden

Belin and Kawahara present a non-technical, practically oriented description of the well-known STRAIGHT algorithm for manipulating and morphing voice recordings, which is now made freely available as a collection of Matlab functions. This is a highly specialized tool, but it has nevertheless proved very useful for research on voice perception, as the authors clearly demonstrate in the text. The availability of this user guide will no doubt make the tool more accessible and help to popularize it further. I have nothing to criticize in the paper itself. Merely as a suggestion for future work, I would consider using an open-source framework (Python, R, Julia, ...) instead of Matlab. Another obvious extension is to add functionality for modifying the source and/or filter after their separation. The filter can be arbitrarily warped (cf. phase vocoders like CLEESE, DAVID etc.), and the source can easily be resynthesized prior to being recombined with the filter for ultimate control (cf. soundgen).

Is the rationale for developing the new software tool clearly explained?

Yes

Is the description of the software tool technically sound?

Yes

Are sufficient details of the code, methods and analysis (if applicable) provided to allow replication of the software development and its use by others?

Yes

Is sufficient information provided to allow interpretation of the expected output datasets and any results generated using the tool?

Yes

Reviewer Expertise:

Human nonverbal vocalizations, voice analysis and synthesis, auditory attention

I confirm that I have read this submission and believe that I have an appropriate level of expertise to confirm that it is of an acceptable scientific standard.

# Reviewer report: Reviewer response for version 1

**DOI:** 10.21956/openreseurope.19511.r42894

**Published:** 2024-09-10

**Authors:** Yi-Wen Liu

**Affiliations:** 1 National Tsing Hua University, Hsinch, Taiwan

As also indicated by the first reviewer, this article is written with clarity and users should be able to follow the tutorials and start using the tool. The applications in Fig. 3 (16-voice morphing) and Fig. 4 (non-primate vocalization) are particularly intriguing to me. With the clever design of user interface which allows user to work intuitively with mObjects, I believe this tool will impact the field and encourage more researchers to try morphing voices for their projects.

 Below are a few editorial suggestions (for the printed version):

 1. In the paragraph Legacy STRAIGHT: the apostrophe seem to be misplaced before the word spectrogram ('spectrogram)

 2. to try and correct -> "to try to correct", or "to correct"?

 3. The term "time axes" is a bit puzzling to me. Does it mean "time location" or simply "time anchor"? I currently assume that a "time axis" is one of the white vertical dotted lines on Fig. 1D and 1E. 

 4. "likely result in unnatural sounding stimuli..." -> remove the trailing periods "..."

 5. In the paragraph "Generating the N-average", what does the abbreviation TVA stand for?

Is the rationale for developing the new software tool clearly explained?

Yes

Is the description of the software tool technically sound?

Yes

Are sufficient details of the code, methods and analysis (if applicable) provided to allow replication of the software development and its use by others?

Yes

Is sufficient information provided to allow interpretation of the expected output datasets and any results generated using the tool?

Yes

Reviewer Expertise:

audio and speech signal processing, hearing science

I confirm that I have read this submission and believe that I have an appropriate level of expertise to confirm that it is of an acceptable scientific standard.

# Response

**Published:** 2024-12-11

**Authors:** Pascal Belin

Many thanks for your positive evaluation.

# Reviewer report: Reviewer response for version 1

**DOI:** 10.21956/openreseurope.19511.r42639

**Published:** 2024-07-31

**Authors:** Nadine Lavan

**Affiliations:** 1 Queen Mary University of London, London, England, UK

This is a clearly written article, describing the voice morphing tool STRAIGHTMORPH.

 From my reading of the paper, all the steps required to morph voices (and other sounds) are described clearly and in an easy-to-follow way. The toolbox is easily accessible via links provided in the paper. Overall, STRAIGHTMORPH is a useful resource for the voice research community.

 I have therefore only one comment: Voice morphing has been used quite widely (in the form of parameter-specific morphing or otherwise) to answer many different research questions. The authors across the manuscript list a number of different papers that have used morphing (e.g. in the section "legacy STRAIGHT") and even provide some details on a small number of studies that have used morphing (see "Morphing Examples"). I however wonder whether a reader who has not encountered voice morphing before as a technique will be able to get a good idea of how versatile voice morphing can indeed be. I believe that adding some additional examples/use cases of how voice morphing has been used before in a bit more detail in the earlier sections might make this paper more impactful overall.

Is the rationale for developing the new software tool clearly explained?

Yes

Is the description of the software tool technically sound?

Yes

Are sufficient details of the code, methods and analysis (if applicable) provided to allow replication of the software development and its use by others?

Yes

Is sufficient information provided to allow interpretation of the expected output datasets and any results generated using the tool?

Yes

Reviewer Expertise:

Voice perception

I confirm that I have read this submission and believe that I have an appropriate level of expertise to confirm that it is of an acceptable scientific standard, however I have significant reservations, as outlined above.

# Response

**Published:** 2024-12-11

**Authors:** Pascal Belin

Thank you for your positive evaluation and your valuable suggestion: readers are now referred to voice morphing examples beyond those described in the manuscript.
